# Supplementary material for: In Vitro Metabolism Study of Seongsanamide A in Human Liver Microsomes Using Non-Targeted Metabolomics and Feature-Based Molecular Networking
Source: Pharmaceutics. 2021 Jul 7;13(7):1031. doi: 10.3390/pharmaceutics13071031 (PMC8309059; doi:10.3390/pharmaceutics13071031)
Supplement: Supplementary file 1 [file pharmaceutics-13-01031-s001.zip › pharmaceutics-1268552-supplementary.pdf]

pharmaceutics

MDPI

Supplementary Materials: In vitro Metabolism Study of Seongsanamide A in Human Liver Microsomes Using Non-Targeted Metabolomics and Feature-Based Molecular Networking

Zhexue Wu, Geum-Jin Kim, So-Young Park, Jong-Cheol Shon, Kwang-Hyeon Liu and Hyukjae Choi

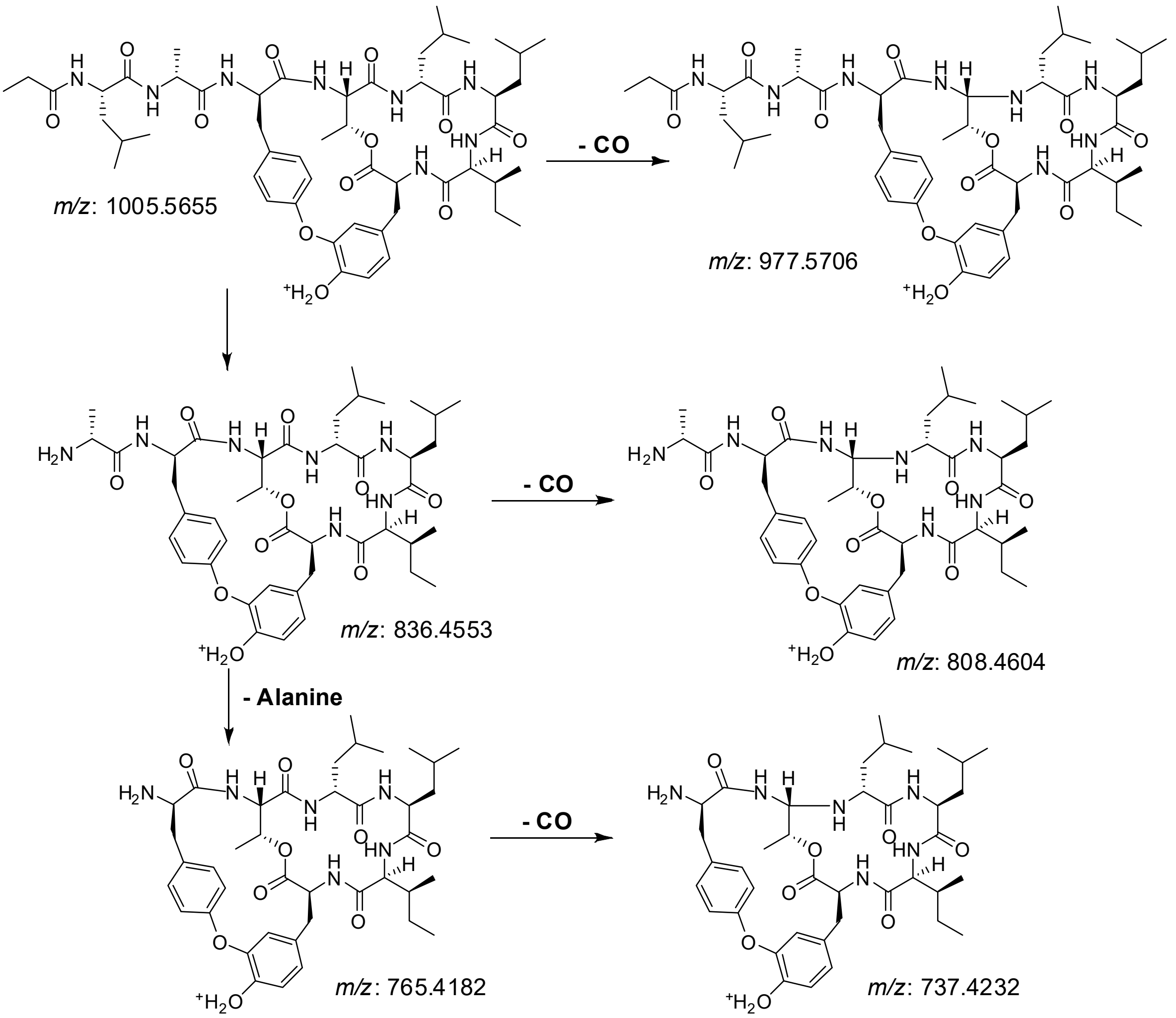

Figure S1. MS/MS fragmentation scheme for seongsanamide A.

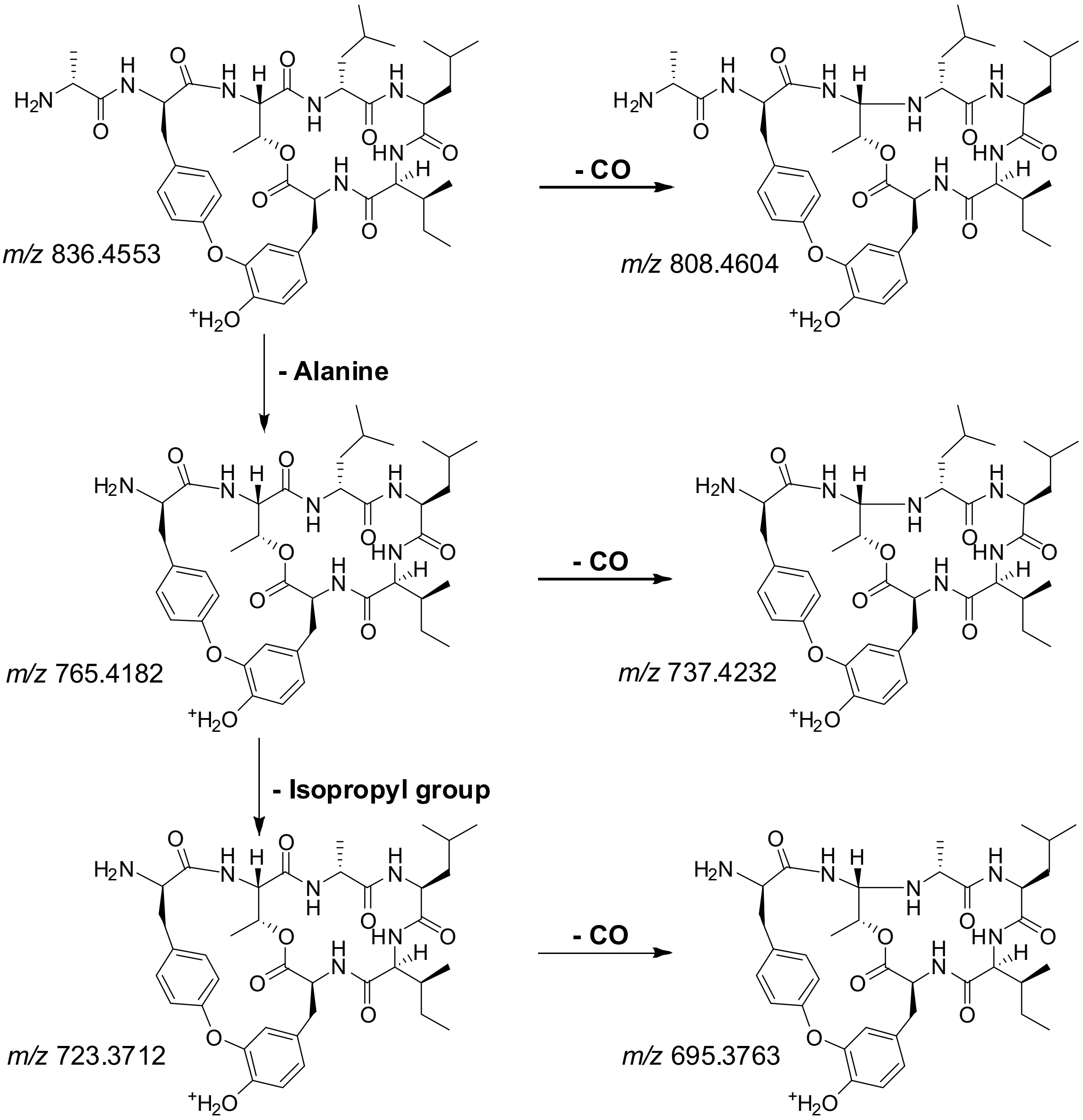

Figure S2. MS/MS fragmentation scheme for hydrolyed seongsanamide A (M1).

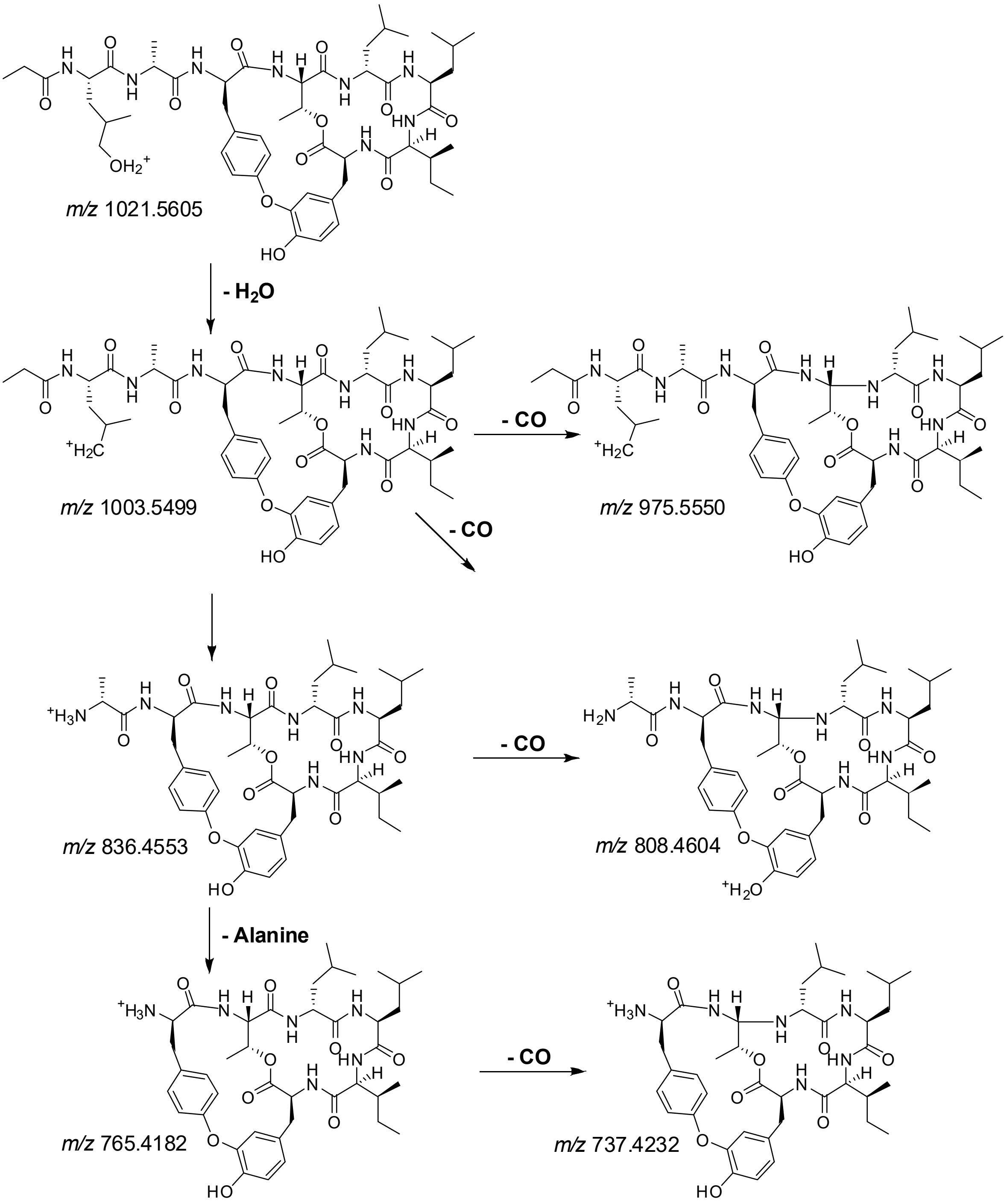

Figure S3. MS/MS fragmentation scheme for monohydroxyseongsanamide A (M2).

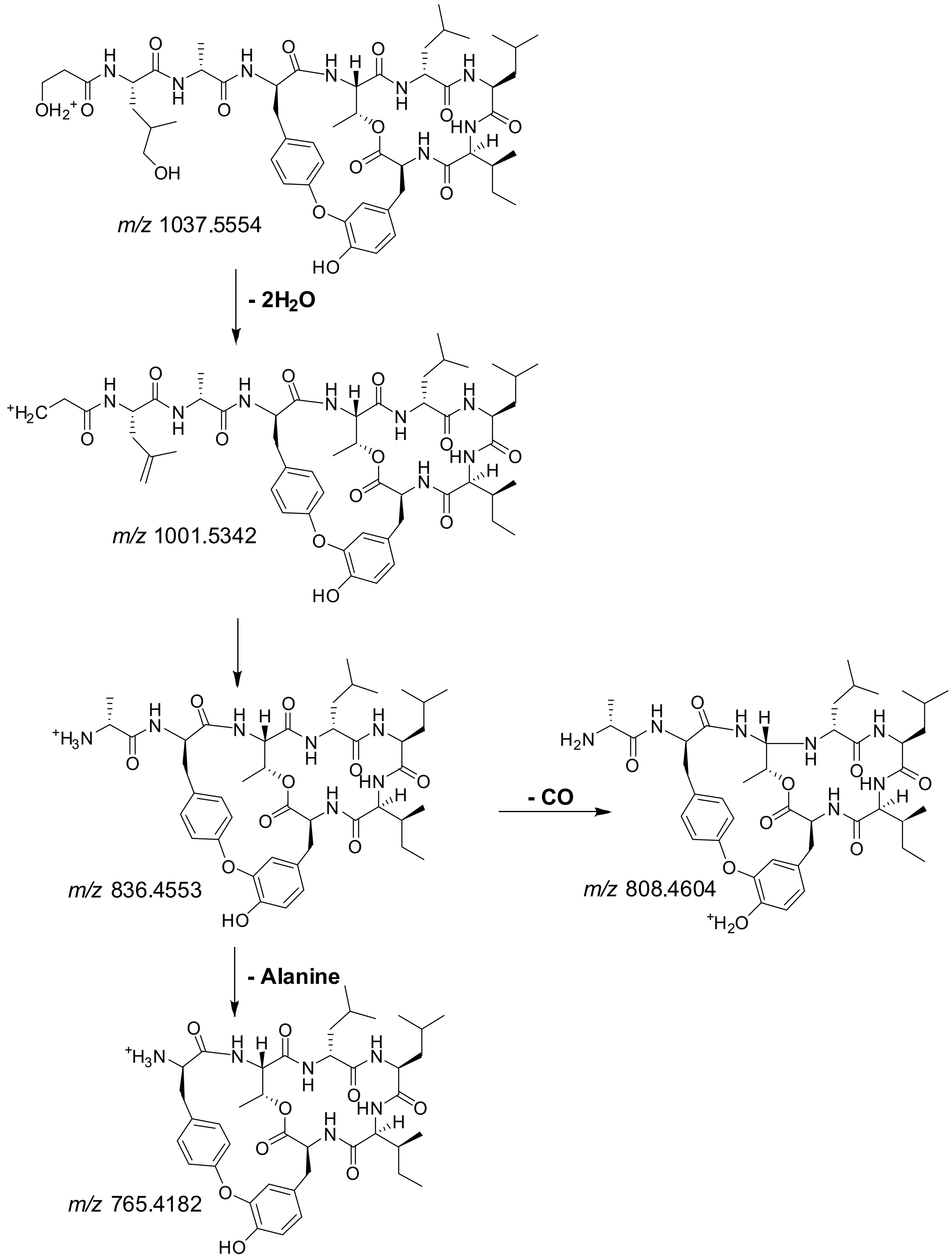

Figure S4. MS/MS fragmentation scheme for dihydroxyseongsanamide A (M3).

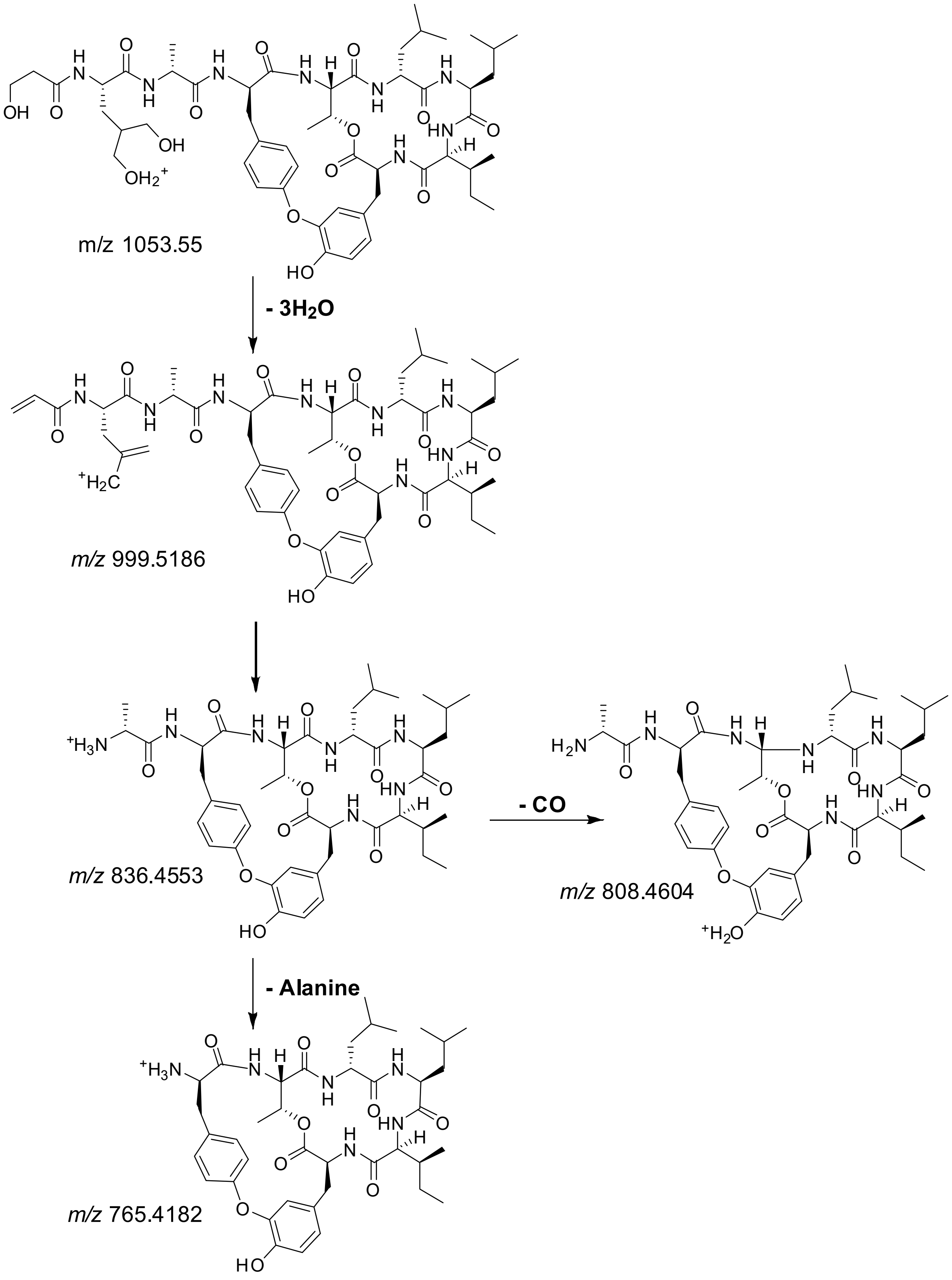

Figure S5. MS/MS fragmentation scheme for trihydroxyseongsanamide A (M4).
